# The Burden of Eclampsia: Results from a Multicenter Study on Surveillance of Severe Maternal Morbidity in Brazil

**DOI:** 10.1371/journal.pone.0097401

**Published:** 2014-05-13

**Authors:** Juliana C. Giordano, Mary A. Parpinelli, Jose G. Cecatti, Samira M. Haddad, Maria L. Costa, Fernanda G. Surita, Joao L. Pinto e Silva, Maria H. Sousa

**Affiliations:** 1 Department of Obstetrics and Gynecology, School of Medical Sciences, University of Campinas - UNICAMP, Campinas, São Paulo, Brazil; 2 Center for Studies on Reproductive Health of Campinas (CEMICAMP), Campinas, São Paulo, Brazil; CUNY, United States of America

## Abstract

**Objective:**

Maternal mortality (MM) is a core indicator of disparities in women’s rights. The study of Near Miss cases is strategic to identifying the breakdowns in obstetrical care. In absolute numbers, both MM and occurrence of eclampsia are rare events. We aim to assess the obstetric care indicators and main predictors for severe maternal outcome from eclampsia (SMO: maternal death plus maternal near miss).

**Methods:**

Secondary analysis of a multicenter, cross-sectional study, including 27 centers from all geographic regions of Brazil, from 2009 to 2010. 426 cases of eclampsia were identified and classified according to the outcomes: SMO and non-SMO. We classified facilities as coming from low- and high-income regions and calculated the WHO’s obstetric health indicators. SPSS and Stata softwares were used to calculate the prevalence ratios (PR) and respective 95% confidence interval (CI) to assess maternal characteristics, clinical and obstetrical history, and access to health services as predictors for SMO, subsequently correlating them with the corresponding perinatal outcomes, also applying multiple regression analysis (adjusted for cluster effect).

**Results:**

Prevalence of and mortality indexes for eclampsia in higher and lower income regions were 0.2%/0.8% and 8.1%/22%, respectively. Difficulties in access to health care showed that ICU admission (_adj_PR 3.61; 95% CI 1.77–7.35) and inadequate monitoring (_adj_PR 2.31; 95% CI 1.48–3.59) were associated with SMO.

**Conclusions:**

Morbidity and mortality associated with eclampsia were high in Brazil, especially in lower income regions. Promoting quality maternal health care and improving the availability of obstetric emergency care are essential actions to relieve the burden of eclampsia.

## Introduction

Eclampsia is a rare, however potentially life-threatening complication of the hypertensive disorders (HD) of pregnancy, accountable for large numbers in morbidity and deaths among women of reproductive age and their offspring [1]–[4]. The estimate of incidence and the burden of eclampsia is still a challenging pursuit worldwide; currently only seven countries have national data on the topic [5]. A systematic review on preeclampsia (PE) and eclampsia, performed in 2013, indicated that the crude incidence of eclampsia fluctuates from 0 to 0.1% in Europe and up to 4% in Nigeria; Brazilian studies showed a 0.6% incidence [5], [6]. Nonetheless, 94.6% of the data were collected in the USA, highlighting a marked regionalization bias and, therefore, the need for more studies, especially in low- and middle-income countries (LMIC) [5], [7].

The case fatality rate (number of deaths/number of cases) of eclampsia ranges from 0–1.8% in high-income countries up to 17.7% in India, emphasizing a huge gap in the quality of maternal health care according to social and economic patterns [8]. Over a one-year period, the Swedish Medical Birth Register identified no maternal death due to eclampsia, whilst in India, in the same period, only one hospital reported 11 eclampsia-related deaths [8]–[10].

Reducing maternal mortality (MM) by three quarters is one of the United Nations’ Millennium Development Goals [11]. Nearly the totality of women who die from pregnancy-related causes comes from LMIC [2], [3]. According to the Brazilian Ministry of Health, there has been a substantial reduction of maternal deaths (MD) in the country from 1990 to 2010, i.e., a decrease from 141 to 62 deaths for 100,000 live births (LB) [12]. Nevertheless, in order to achieve the MDG5 by 2015, Brazil would have to halve this number, what seems to be a very difficult mission to pursue.

Recently, the World Health Organization (WHO) defined the presence of organ dysfunction or failure during pregnancy, childbirth or postpartum as maternal near miss (NM). A woman who fulfills one of the clinical, laboratory or management criteria established by WHO is a NM case. From a theoretical perspective, the NM cases should be as similar to maternal deaths as possible [13]–[17].

Childbirth care in LMIC is usually associated with difficult access to adequate maternity services [2], [3], [7]. In Brazil, although 98% of pregnant women do deliver their babies in hospitals, a large number of these facilities are not well equipped to deal with pregnancy-related complications. The shortage of intensive care units (ICU) to where such women can be transferred is still a worrying reality in several settings [12]. In addition the proportion of facilities with adequately trained staff to deal with complications is not known at all.

MM is amongst the worst-performing health indicators in resource-poor settings. In absolute numbers, both maternal mortality and the occurrence of eclampsia are rare events [2], [3], [16], [17]. The only Brazilian national data on eclampsia is the total number of deaths, 167 cases in 2010, with a maternal mortality ratio (MMR) of 5.83 [18]. It is with the intent of filling this epidemiological gap that our study aims to assess the obstetric care indicators and main predictors for severe maternal outcomes from eclampsia (SMO: maternal death plus maternal near miss).

## Methods

Our study is a secondary analysis of The Brazilian Network for Surveillance of Severe Maternal Morbidity Study. The purpose of this network was to identify cases of severe maternal morbidity/near miss, using the criteria recently established by WHO to characterize these conditions [16]. According to this definition, a maternal near miss case is a woman who experienced a very serious complication during pregnancy and as a consequence almost died, surviving at least until the 42^nd^ day after childbirth. The methods of the Brazilian Network have already been described in details elsewhere [19], [20].

Briefly, it was a cross-sectional multicenter study conducted from July 2009 to June 2010, involving 27 hospitals from all different regions of Brazil, excluding the Federal District. From those 27 centers, 95% of cases were insured by SUS, the Brazilian publicly funded health system. Brazil is geographically divided into 5 different regions and one Federal District: North (N), Northeast (NE), Midwest (MW), South (S) and Southeast (SE). We assembled these regions into 2 major groups, according to their 2000, human development index (HDI) [21]. According to this definition, S and SE were high HDI regions and N, NE and MD were low HDI regions. We then calculated the indicators proposed by WHO to monitor the quality of obstetric care using maternal near miss and maternal death cases with eclampsia [16].

During this period, out of the 9,555 women who were diagnosed with severe maternal complications, 6,706 presented with severe hypertensive disorders and 426 were admitted with, or developed, eclampsia during hospitalization. Eclampsia was identified by the occurrence of tonic-clonic seizures - including seizures and coma - that occurred during pregnancy, delivery or puerperium and that were not related to preexisting organic brain disorders [1].

### Main Outcomes

Maternal outcomes for eclampsia during pregnancy, childbirth or puerperium were considered in two different groups:

#### Non-Severe Maternal Outcome (non-SMO)

All cases of eclampsia in the absence of organ failure/dysfunction were classified as non-SMO; this is the comparison group.

#### SMO (Severe maternal outcome)

All cases of maternal death or maternal near miss.

**Figure 1:**
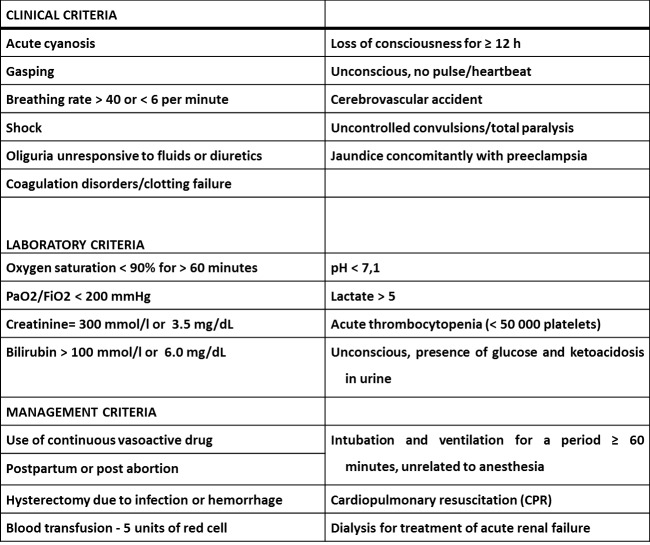
WHO criteria for maternal near miss.

- *Maternal Near Miss (NM)*: cases that fulfilled at least one of the clinical, laboratory or management criteria representing life-threatening conditions (i.e., organ failure/dysfunction) and who survived this condition. Figure 1.
- *Maternal Death (MD)*: death during pregnancy or within 42 days post-partum, regardless length or site of pregnancy, from any cause related to or aggravated by the pregnancy or its management, yet not from accidental or incidental causes.

### Covariates

Information on age, skin color, marital status, schooling and parity were analyzed as possible predictors of SMO from eclampsia, as they are already been identified in some studies as predictors for eclampsia [4], [22], [23].

Previous disease was defined as any pathological condition diagnosed before or during pregnancy, but not related to it. Chronic hypertension was defined as the presence of high blood pressure (BP) ≥140×90 mmHg diagnosed before the 20^th^ week of pregnancy, after two measurements within a minimum interval of 4 hours, regardless of the use of medications [1].

We selected the most frequent associated complications during admission period: hemorrhage, HELLP syndrome, severe hypertension, pulmonary edema and severe sepsis. Coagulation disorders, shock, jaundice concomitantly with preeclampsia and cerebrovascular accident are part of the NM definition criteria, and were therefore excluded from the analysis because of their behavior as interacting variables.

The use of magnesium sulphate (MgSO_4_) for the prevention and management of eclampsia was assessed as a dichotomous variable (use and non-use) because the data collection form had no information on the exact period of time when clinical events occurred or procedures were performed, Therefore the opportunity of its use could not be detailed assessed.

Post-partum admission was regarded as a worse outcome, based upon the assumption that the eclamptic women who were admitted after giving birth had to be transferred to a health center capable of delivering a better care. Bearing in mind that not all the Brazilian health facilities caring for pregnant women are equipped with an ICU or have an ICU bed promptly available (most ICUs operate at their full capacity at any given time), we had to assume that only the most complicated cases of eclampsia were admitted to ICU. In addition to this inference, ICU availability was also assessed by the variable “inadequate monitoring”, translated into availability or not of ICU care.

Our study had local coordinators who were trained to gather accurate information from both health care providers involved with the care at its initial phases and from medical records, aiming to address as many aspects of care as possible. We classified and defined the variables “lack of drug”,^ “^inadequate monitoring”, “delay for transfer”, “lack of staff”, “delay for diagnosis”, “not opportune treatment” and “inadequate management” to evaluate the access to and quality of appropriate obstetric emergency care. This was performed by both the local investigator and coordinator and then checked by the study team of the coordinating center.

We finally analyzed the perinatal outcomes and mode of delivery in SMO cases. The variables were defined as follows: cut-off point for gestational age at delivery, determined by clinical criteria (less than 33 weeks and ≥34 weeks); mode of delivery, listed as cesarean section or vaginal birth; perinatal outcome (stillbirth or live birth); birth weight (<2500 or ≥2500 grams); neonatal outcome (defined as neonatal ICU admission, neonatal death, i.e., death until 28 days of life, or hospital discharge); fifth minute Apgar score (<7 and from 7 to 10 indicates, respectively low and high vitality score at birth); and perinatal death (stillbirth plus neonatal death <7 days).

### Statistical Analysis

Bivariate analysis was performed to identify factors (predictors) associated with SMO (maternal NM or MD) by estimating prevalence ratios (PR) and their respective 95% confidence intervals (CI), adjusted for cluster effect (maternal hospital or centers) [24]. Access to health care facilities, maternal characteristics, complications and procedures related to and/or used for management of eclampsia, other than those already used for NM case definition according to the WHO criteria, were described comparatively among women from both groups, with differences assessed by a Chi-square test. Additionally the hazard of perinatal outcomes including the mode of delivery was also estimated for women who progressed towards an SMO, with adjusted PR and their respective 95% confidence interval (CI). Finally, Poisson multiple regression analysis was performed in 321 cases in which all variables were available and adjusted by cluster and all other predictors. The primary sampling units of our study were the health care facilities and therefore it was necessary to adjust the analysis by the cluster effect [25]. The softwares used for the analysis were SPSS version 17 (SPSS, Chicago, IL, USA) and Stata version 7.0 (StataCorp, College Station, TX, USA).

### Quality Control

The network database was fed with information extracted from the medical records, transcribed manually onto the data collection form by local investigators and later on transferred to the electronic forms. The technical procedures for case selection and accurate form filling were detailed explained in the respective manual of operation. Local study coordinators performed systematic quality control of data, so that possible incongruences could be identified. One of the investigators from the coordinating center visited the institutions taking part of this study, aiming to verify the consistency of the information retrieved from both manually- and electronically-filled collection forms in light of the case reports of the study subjects, randomly selecting such cases. The final quality control was performed by the application of logical consistency and review of database.

### Ethical Statement

This study is a secondary analysis; all records were obtained through the database of the main study, the Brazilian Network for Surveillance of Severe Maternal Morbidity. According to the rules of the sponsor agency the database is not of public domain and the principal investigators are the owners of the data, being responsible for its use for scientific purposes. We followed all the principles that regulate research on human beings defined by the Brazilian National Health Council, as well as the Declaration of Helsinki. There was no need for an Informed Consent Form, since data were collected from medical records post–discharge or post-mortem and no contact occurred with the subjects. Local IRBs (listed below) and the National Committee of Ethics in Research (CONEP, Brazilian Ministry of Health) approved the study, under the letter of approval 097/2009. The National Council for Ethics in Research and the Institutional Review Boards of each site granted a waiver of individual informed consent.

The Review Boards of the following institutions reviewed and approved this study: Maternidade Cidade Nova Dona Nazarina Daou (Manaus, AM), Maternidade Climério de Oliveira (Salvador, BA), Hospital Geral de Fortaleza (Fortaleza, CE), Hospital Geral Dr. César Cals (Fortaleza, CE), Maternidade Escola Assis Chateaubriand (Fortaleza, CE), Hospital Materno Infantil de Goiania (Goiania, GO), Hospital Universitário da Universidade Federal do Maranhao (Sao Luis, MA), Maternidade Odete Valadares (Belo Horizonte, MG), Instituto de Saúde Elıdio de Almeida (Campina Grande, PB), Hospital Universitário Lauro Wanderley da Universidade Federal da Paraiba (Joao Pessoa, PB), Centro Integrado de Saúde Amaury de Medeiros (Recife, PE), Instituto de Medicina Integral Prof. Fernando Figueira (Recife, PE), Hospital das Clınicas da Universidade Federal de Pernambuco (Recife, PE), Hospital das Clınicas da Universidade Federal do Paraná (Curitiba, PR), Hospital Maternidade Fernando Magalhaes (Rio de Janeiro, RJ), Instituto Fernandes Figueira (Rio de Janeiro, RJ), Hospital das Clinicas da Universidade Federal do Rio Grande do Sul (Porto Alegre, RS), Faculdade de Medicina de Botucatu da Universidade Estadual Paulista (Botucatu, SP), Hospital da Mulher da Universidade Estadual de Campinas (Campinas, SP), Hospital e Maternidade Celso Pierro da Pontifıcia Universidade Católica (Campinas, SP), Hospital Israelita Albert Einstein (São Paulo, SP), Faculdade de Medicina de Jundiaí (Jundiaı, SP), Hospital das Clınicas da Faculdade de Medicina de Ribeirão Preto da Universidade de São Paulo (Ribeirão Preto, SP), Santa Casa de Limeira (Limeira, SP), Santa Casa de São Carlos (São Carlos, SP), Casa Maternal Leonor Mendes de Barros (São Paulo, SP), Hospital São Paulo da Universidade Federal de São Paulo (São Paulo, SP).

## Results

**Figure 2:**
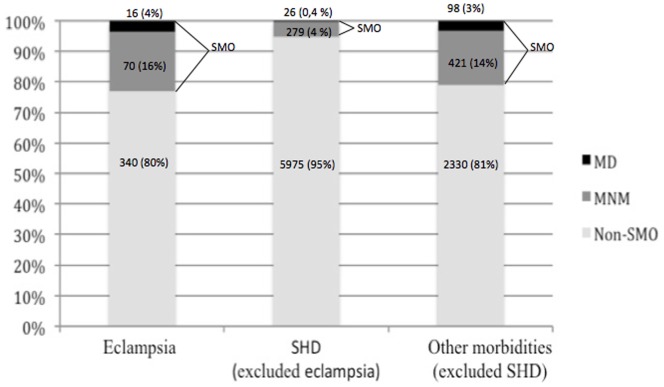
Distribution of Non-SMO, MNM and MD in women with eclampsia, others SHD and other morbidities [Non-SMO = women without severe maternal outcomes (MNM or MD), SMO = Severe maternal outcome (MNM = maternal near miss or MD = maternal death), SHD = severe hypertensive disorders].

In the one-year study period, there were 82,388 women admitted to the 27 maternity hospitals participating in the study; these women gave birth to 82,144 live births (LB). 9,555 women presented pregnancy-related severe complications and met the study inclusion criteria. Out of this population, 910 women progressed to SMO (770 maternal NM and 140 maternal deaths); 20% of cases of eclampsia, 4% of cases of other severe hypertensive disorders (excluding eclampsia) and 17% of other morbidities (infectious and hemorrhagic disorders) developed SMO. Respectively, almost 4% of cases of eclampsia and other morbidities and 0.4% of cases of other severe hypertensive disorders died. (Figure 2).

The total prevalence of eclampsia was 5.2 (per 1000 LB) and a specific MMR of 19.5 (per 100,000 LB). The total mortality index was 18.6%, 2.7 times higher in the Brazilian regions of lower HDI: 22.6% gathering the Midwest, Northeast and North regions, and 8.3% for South and Southeast regions (Table 1).

**Table 1:** Obstetric health indicators for eclampsia to total of cases and according to the level of income from the Brazilian regions where the facilities are located.

Obstetric Care Indicators	LI Regions N/NE/MW	HI Regions S/SE	Total
**Maternal Near miss**	48	22	70
**Maternal Death**	14	2	16
**Live Births**	39,747	42,397	82,144
**SMO**	62	24	86
**NMM ratio/1000 LB**	1.2	0.5	0.85
**SMO ratio/1000 LB**	1.55	0.56	1.04
**NM: MD**	3.4∶1	11∶1	4.35∶1
**MDI (%)**	22.6	8.33	18.6
**Prevalence of eclampsia per 1000 LB**	8.37	2.2	5.18

SMO = severe maternal outcome, NMM = Maternal Near miss, LB = live births, MDI = maternal death index (MDI = MD/MD+NM), LI: low income, HI: high income.

Approximately 70% of all eclamptic women were primiparous. The median age of the cases was 20 years, with the youngest being 13 and the eldest 44 (data not showed in tables). Amongst the maternal characteristics, obstetric background and medical history, the only factors associated with the risk of SMO from eclampsia were any previous disease and chronic hypertension. Medical histories of any previous disease were present in 27% of the cases and almost doubled the risk of SMO (PR 1.86; CI 1.35–2.57) (Table 2).

**Table 2:** Rate of SMO and estimated risk of SMO for eclampsia according to maternal characteristics, obstetric background and medical history.

Characteristics	SMO rate	N %	Non-SMO	N %		PR	95% CI
**Age (years)**							
≤19	33	16.4	168		83.6	0.73	0.42–1.28
20–34	45	22.5	155		77.5	Ref.	
≥35	8	32.0	17		68.0	1.42	0.89–2.27
**Ethnicity (a)**							
Non white	34	19.3	142		80.7	0.92	0.56–1.53
White	23	20.9	87		79.1	Ref.	
**Marital Status (b)**							
Without partner	23	14.9	131		85.1	0.78	0.52–1.17
With partner	35	19.2	147		80.8	Ref.	
**Schooling (c)**							
Elementary	21	14.2	127		85.8	1.00	0.46–2.17
> Elementary	19	14.2	115		85.8	Ref.	
**Number of pregnancies (d)**							
1	52	17.7	242		82.3	0.75	0.54–1.05
>1	29	23.6	94		76.4	Ref.	
**Any previous disease (e)**							
Yes	25	27.2	67		72.8	**1.86**	**1.35–2.57**
No	36	14.6	211		85.4	Ref.	
**Chronic hypertension (e)**							
Yes	10	30.3	23		69.7	**1.82**	**1.14–2.90**
No	51	16.7	255		83.3	Ref.	

SMO = severe maternal outcome (maternal near miss plus maternal death), PR: prevalence ratio adjusted by cluster effect, CI: confidence interval.

Missing data for: (a) 140 cases, (b) 90 cases, (c) 144 cases, (d) 9 cases, (e) 87 cases.

Values in bold mean they are significant.

Moreover, the adequacy of the prenatal care received, indirectly evaluated by the number of visits corrected for gestational age at birth, was appropriate in more than 67% of the total number of cases and the moment of hospital admission, if still during pregnancy (80%) or after giving birth (20%), showed no association with worse outcome amongst eclamptic women (Table 3).

**Table 3:** Rate of SMO and estimated risk of SMO for eclampsia according to several characteristics concerning access to health care facilities.

Characteristics	SMO rate	N %	Non-SMO	N %	PR	95% CI
**Prenatal adequacy (a)**						
no	1	5.9	16	94.1	0.34	0.06–1.96
yes	49	17.1	237	82.9		
**Post-partum admission**						
yes	22	26.8	60	73.2	1.44	0.97–2.15
no	64	18.6	280	81.4		
**ICU admission**						
yes	73	31.4	159	68.6	**4.70**	**2.81–7.84**
no	13	6.7	181	93.3		
**MgSO_4_ prescription**						
no	5	35.7	9	64.3	1.82	0.79–4.20
yes	81	19.7	331	80.3		
**Lack of drug (b)**						
yes	9	27.3	24	72,7	0.83	0.83–2.45
no	68	19.1	288	80.9		
**Inadequate monitoring (b)**						
yes	21	47.7	23	52.3	**2.94**	**2.13–4.07**
no	56	16.2	289	83.8		
**Delay for transfer (b)**						
yes	12	40.0	18	60.0	**2.32**	**1.33–4.05**
no	62	17.3	297	82.7		
**Lack of staff (b)**						
yes	17	33.3	34	66.7	**1.88**	**1.20–2.93**
no	60	17.7	278	82.3		
**Delay for diagnosis (b)**						
yes	19	37.2	32	62.8	**2.29**	**1.42–3.69**
no	55	16.3	283	83.7		
**Treatment opportunity (b)**						
no	24	35.3	44	64.7	**2.27**	**1.48–3.46**
yes	50	15.6	271	84.4		
**Inadequate management (b)**						
yes	28	29.2	68	70.8	**1.86**	**1.33–2.60**
no	46	15.7	247	84.3		

SMO = severe maternal outcome (maternal near miss and maternal death), PR: prevalence ratio adjusted by cluster effect, CI: confidence interval, MgSO_4_: magnesium sulphate.

Missing data from: (a) 123 cases, (b) 37 cases.

Values in bold mean they are significant.

Variables used to evaluate the access to health care demonstrated a robust association with the risk of SMO from eclampsia (Table 3). ICU admission (PR 4.70; 95% CI 2.81–7.84), inadequacies of monitoring (PR 2.94; 95% CI 2.13–4.07); delay for transfer (PR 2.32; 95% CI 1.33–4.05); lack of trained staff (PR 1.88; 95% CI 1.20–2.93); delay for diagnosis (PR 2.29; 95% CI 1.42–3.69), not opportune treatment (PR 2.27; 95% CI 1.48–3.46) and inadequate management of the case (PR 1.86; 95% CI 1.33–2.60) led to a two- to four-fold increased in the risk of SMO.

Poisson multiple regression analyses confirmed admission to ICU, lack adequate monitoring, severe sepsis and any previous disease as the main independent predictors for SMO. All complications of pregnancy were strongly associated with increased risk of SMO (data not presented in tables), despite the fact that only severe sepsis, on the multiple regression analysis, predicted SMO (adjusted PR 2.75; 95% CI 1.35–5.61) (Table 4). Nevertheless, the incidence of such complications among eclamptic women was not similar, fluctuating from 1% (pulmonary edema) to 12% (HELLP). (Data not presented in tables.).

**Table 4:** Variables independently associated with severe maternal outcome by Poisson multiple regression analysis (n = 321).

Factors	Adjusted PR*	95% CI	p
**Inadequate Monitoring**	2.31	1.48–3.59	0.001
**ICU admission**	3.61	1.77–7.35	0.001
**Any previous disease**	1.82	1.26–2.64	0.003
**Severe Sepsis**	2.75	1.35–5.61	0.007

*Considering cluster design (center/hospital), PR: prevalence ratio, CI: confidence interval, ICU = intensive care unit.

Statistical model including variables: Age, ethnicity, marital status, schooling, number of pregnancies, any previous disease, chronic hypertension, post-partum admission, ICU admission, magnesium sulphate use, lack of medication, inadequate monitoring, delay in transfer, lack of trained staff, diagnosis delay, treatment opportunity, inadequate management, hemorrhagic complication, HELLP syndrome, severe hypertension, pulmonary edema, severe sepsis, gestational age at birth.

Gestational age at delivery reached a median of 36 weeks, fluctuating from 22 to 42 weeks; birth weight implied a median of 2.410 grams, ranging from 520 to 4.900 grams (data not presented in tables) and C-section accounted for 88% of all deliveries. Perinatal outcomes were also substantially worse in the SMO group: stillbirth (PR 2.34, 95% CI 1.29–4.24), neonatal ICU admission (PR 1.84; 95% CI 1.09–3.10), neonatal death (PR 2.68; 95% CI 1.21–5.91), low 5-min Apgar score (PR 2.87; 95% CI 1.79–4.62) and perinatal death (PR 2.3; 95% CI 1.45–3.65) (Table 5).

**Table 5:** Estimated risk of SMO according to gestational age, mode of delivery and perinatal outcomes in women with eclampsia.

	SMO		Non-SMO			PR	95% CI
	n	%	n		%		
**Gestational age at delivery (a)**							
22 to 33 weeks	24	35.3	84	28.0		1.31	0.80–2.14
≥34 weeks	44	64.7	216	72.0			
**Mode of delivery (b)**							
C-section	76	91.6	290	86.8		1.51	0.74–3.11
Vaginal birth	7	8.4	44	13.2			
**Perinatal outcome (c)**							
Still births	12	16.0	17	5.5		**2.34**	**1.29–4.24**
Live births	63	84.0	293	94.5			
**Birth weight (d)**							
<2500 g	40	62.5	150	50.7		1.49	0.93–2.39
≥2500 g	24	37.5	146	49.3			
**Neonatal outcome (e)**							
NICU admission	24	40.7	74	26.6		**1.84**	**1.09–3.10**
Neonatal death	5	8.5	9	3.2		**2.68**	**1.21–5.91**
Hospital discharge	30	50.8	195	70.1			
**Apgar at 5^th^ min (f)**							
<7	14	24.6	22	7.7		**2.87**	**1.79–4.62**
7 a 10	43	75.4	263	92.3			
**Perinatal death (g)**							
Yes	15	21.7	24	8.2		**2.30**	**1.45–3.65**
No	54	78.3	269	91.8			

SMO = severe maternal outcome (maternal near miss plus maternal death), PR: prevalence ratio adjusted for cluster effect, CI: confidence interval, NICU = neonatal intensive care unit.

Missing data from: (a) 58, (b) 8 missing cases and 1 abortion, (c) 41, (d) 66, (e) 89, (f) 68, (g) 64 cases.

Values in bold mean they are significant.

## Discussion

Our study presents an overview of the clinical morbidities and the access to health care for women with eclampsia in selected obstetric units in the five geographical Brazilian regions. These results confirm a prevalence of SMO for eclampsia five times higher than for other severe hypertensive disorders of pregnancy group (excluding cases of eclampsia). In fact, eclampsia is a major cause of morbidity and death in this group. Multiple regression analysis pointed out that the lack of emergency care facilities in obstetric units are predictors of worse outcomes among women with eclampsia. In addition, any previous disease and severe sepsis were also main predictors of SMO from eclampsia. The higher risk of dying found when ICU admission was present shows that probably there was a pre-selection of most severe cases towards admission to ICU, and the already well-known shortage of ICU beds in many Brazilian health care facilities has a major impact on this result [26].

Three quarters of the Brazilian population are insured by SUS, the Brazilian publicly funded health system, and the remaining one quarter relies on the insurance and/or private health sector. The only center included in the Brazilian Network that provides care exclusively to high-income private patients did not have any case of eclampsia over this one-year period.

Our study has several strengths, including a broad geographical distribution of the participating centers, assuring the representativeness of all regions (with 55.6% LB from the South and Southeast, and 48.4% from the North, Northeast, and Midwest); a rigorous three-step check for control of data quality, as described; and a large sample size due to the eligibility of many of the cases. We found a proportion of 9.5% of SMO for eclampsia among the 910 cases of SMO from the network, very close to that found by the WHO Multicountry Survey (9.6%) undertaken in 29 countries and 357 health facilities, recently published [27].

Figure 1 demonstrates that SMO was present in 20% of cases of eclampsia and was responsible for the majority of SMO in the severe hypertensive disorders group. One of our study’s strengths is the design of a severity scale over which eclampsia cases can be split into two groups. This provides a clear view of the main predictors of worse outcomes. In other words, even though eclampsia is a rare event, the percentage of life threatening complications or death due to it is still extremely high in our population.

Notwithstanding the well-established association between occurrence of eclampsia and maternal characteristics such as age, ethnicity, marital status, years of education and parity, our findings did not identify the same patterns when assessing the risk of SMO from eclampsia [4], [22], [23]. One possible hypothesis is that no matter how robust the association between the occurrence of eclampsia and low Human Development Index (HDI) and its indicators (e.g., low schooling and income) is, once a woman seizures, the outcomes mainly rely on proper and timely care, irrespective of the social and economic background characteristics [7].

All associated complications (hemorrhage, HELLP syndrome, severe hypertension, pulmonary edema and sepsis) were associated with SMO. The study was originally designed to perform a surveillance of severe complications in all pregnancies from the participating institutions during a fixed period of time. Therefore it was a decision to keep the data collection form as short as possible to facilitate its implementation. Thus specific questions to go deeper in each cause or associated factor were decided not to be included. In addition, taking into account the fact that information was cross-sectionally collected after the women was discharged, the information regarding the exact time on each event occurred or each procedure was performed is not available. That is the reason why our study could only assess use and non-use of MgSO_4_ and not its appropriateness. Being a key drug to prevent seizures in situations of severe preeclampsia, we can make the inference that its use on the study population was almost always delayed or inappropriate, as all the included cases had seizures [28], [29].

Fourteen women (3%) did not receive MgSO_4_ at all, which is noteworthy, considering it is a low-cost, effective, life-saving and well-known drug. Our study, however, did not find a significantly increased risk of SMO from eclampsia in cases to whom MgSO_4_ was not administered. The remaining 97% of cases in our study were prescribed MgSO_4_ at any given time, what is a remarkably high figure, especially when compared to the finding of 89% MgSO_4_ use reported in the recently published WHO Multicountry Survey that evaluated 29 different LMIC countries [27]. Only 8% of the cases were reported as having no access to the drug in any given time before or after delivery.

We found a 5-fold increase in the risk of SMO among our population with eclampsia, mostly as a consequence of delays in diagnosis, delay in transportation. inadequate management, lack of well-trained staff and lack of intensive care unit These variables quantify barriers and delays for proper obstetric care, and our findings reinforce studies that pointed those factors out as the main challenges for improving maternal and perinatal health care in LMIC [7], [8], [30], [31]. The 3-fold higher risk of SMO in women that had difficulties in accessing the obstetric ICU corroborates other findings that had already pointed to the need for staff training and better infrastructure for maternal facilities and obstetric ICUs for the delivery of prompt and adequate care to severe obstetric complications [26], [28]. These categorizations were of course attributed with the knowledge of the SMO status. If a risk of information bias is likely to exist, this would probably be in the way of diminishing, and not showing that these substandard care/delays in fact occurred, considering that both local investigators and coordinators were also part of the clinical staff of each participating center.

To the best of our knowledge there are no published findings concerning perinatal outcomes from two different groups of severity (SMO and non-SMO) of eclampsia, making comparisons difficult to be established. We found a 10% total prevalence of perinatal mortality among cases of eclampsia. According to a recent systematic review, a Nigerian study presenting perinatal outcomes in eclampsia treated with MgSO_4_ found 30% incidence of perinatal mortality, and a British one, 6%. Considering the 97% use of magnesium sulphate in our cases, we could argue that our perinatal mortality amongst cases of eclampsia is more similar to that of the UK than to that of Nigeria [32], [33].

Regarding the possible limitations of our study, the database cannot bet understood as representative of the whole Brazilian population. However it had a multicenter cross sectional design and an appropriate sample size. Secondly, some maternal characteristics are challenging to evaluate, for instance, numbers on skin color and years of education were missing in approximately a third of the database, marital status in a fifth - yet those variables did not appear as predictors of SMO. At the same time, the key variables that predicted maternal SMO had less than 10% of missing data. One of the particularities of studying NM cases is the possibility to interview women after life-threatening events, thus identifying breakdowns in health systems [16]. In our study no interviews were undertaken however, but we developed a structured form and trained investigators to gather information on access to care not only from medical records, but also from hospital staff, and we included an open variable that could be used to describe a randomly peculiar characteristic that could not have been contemplated by the form. Thus we were able to include insights on health systems problems. As examples we could quote: “after C-section in Cabedelo city a patient was transferred to state capital Joao Pessoa (25 km) for ICU admission at 7 pm, and died after several seizures at 9 pm”; or “MgSO_4_ administered after C-section, patient had other seizure after procedure.”

Brazil is a member of the BRICS nations group, which also includes Russia, India, China and South Africa. The current economic up growth, combined with a significant influence on regional and global matters, bond these emerging nations. It is well known that social and educational improvements do not always progress hand-in-hand with the economic boom, and this is still a challenge not only for Brazil but for the whole BRICS community.

In conclusion, improvements in social and educational structures alone will probably not lead to the needed changes on time for the Millennium Development Goal number 5 to be achieved by 2015. Our findings point out clearly that lower income regions in Brazil have a worse performance in all obstetric health care indicators among women with eclampsia. The strengthening of health systems might be a possible strategy to reduce morbidity and deaths in women of reproductive age and their offspring [28], [34]. It is known that social and economic determinants are associated with higher maternal and perinatal mortality [3], [32]. Waiting for changes in those patterns in order to get better obstetric and perinatal outcomes might not be the faster route to reduce SMO due to eclampsia. Instead, qualifying emergency obstetric health care by promoting continued staff training and increasing the number of well-equipped health care facilities (especially obstetric ICU beds) are a more plausible and expedient pathway not only for Brazil, but also for all other LMIC and emerging nations who endeavor to relieve the burden of eclampsia.
