# Correction: The Burden of Eclampsia: Results from a Multicenter Study on Surveillance of Severe Maternal Morbidity in Brazil

**DOI:** 10.1371/journal.pone.0102208

**Published:** 2014-07-01

**Authors:** The PLOS ONE Staff

## Notice of Republication

This article was republished on June 10, 2014, to replace copyrighted or confidential material and to update the funding statement. Please download the article again to view the corrected version.
